# TGF-Beta as a Master Regulator of Diabetic Nephropathy

**DOI:** 10.3390/ijms22157881

**Published:** 2021-07-23

**Authors:** Li Wang, Hong-Lian Wang, Tong-Tong Liu, Hui-Yao Lan

**Affiliations:** 1 Research Center for Integrative Medicine, Affiliated Traditional Medicine Hospital of Southwest Medical University, Luzhou 646000, China; wangli120@swmu.edu.cn (L.W.); hackie_wang@126.com (H.-L.W.); tongtong9408@126.com (T.-T.L.); 2 Department of Medicine and Therapeutics, and Li Ka Shing Institute of Health Sciences, Chinese University of Hong Kong, Hong Kong 999077, China

**Keywords:** TGF-β signaling, diabetic nephropathy, Smad, fibrosis, inflammation

## Abstract

Diabetic nephropathy (DN) is one of the most common complications in diabetes mellitus and the leading cause of end-stage renal disease. TGF-β is a pleiotropic cytokine and has been recognized as a key mediator of DN. However, anti-TGF-β treatment for DN remains controversial due to the diverse role of TGF-β1 in DN. Thus, understanding the regulatory role and mechanisms of TGF-β in the pathogenesis of DN is the initial step towards the development of anti-TGF-β treatment for DN. In this review, we first discuss the diverse roles and signaling mechanisms of TGF-β in DN by focusing on the latent versus active TGF-β1, the TGF-β receptors, and the downstream individual Smad signaling molecules including Smad2, Smad3, Smad4, and Smad7. Then, we dissect the regulatory mechanisms of TGF-β/Smad signaling in the development of DN by emphasizing Smad-dependent non-coding RNAs including microRNAs and long-non-coding RNAs. Finally, the potential therapeutic strategies for DN by targeting TGF-β signaling with various therapeutic approaches are discussed.

## 1. Introduction

Diabetic nephropathy (DN) is a common complication in patients with diabetes mellitus (diabetes hereafter) and a major cause of chronic kidney disease (CKD) and end-stage renal disease (ESRD). DN is characterized by the development of proteinuria (microalbuminuria), mesangial cell proliferation and matrix expansion, and glomerular and tubulointerstitial fibrosis [1]. In the early stage of DN, the glomerular filtration rate (GFR) can be increased [2], but finally declines as the consequence of progressive renal injury [1].

Both type 1 and type 2 diabetes can cause DN but not all patients with diabetes eventually develop DN. The incidence of DN in the American population with diabetes is 20–40%; of this group, a considerable portion of patients can preserve the normal renal function for a long period postdiagnosis of diabetes [1]. This strongly suggests that genetic predisposition may be an important risk factor in the onset of DN, which has been validated by a large cohort-based GWAS study [3]. It has been shown that single nucleotide polymorphism (SNP) at codon 10 (Pro10Leu) and codon 263 (Thr263Ile) of *Tgfb1* is associated with the development of nephropathy in type 1 diabetes [4,5]. The association of Pro10Leu with DN in type 2 diabetes is also reported in the Chinese population [6]. *In vitro*, the genetic polymorphism of *Tgfb1* may influence its expression [7], although this is not supported by other epidemiological studies [8,9]. In patients with type 1 and type 2 diabetes, plasma levels of TGF-β1 increase significantly and become further elevated in those with DN [10,11,12,13]. Correlation analysis shows that the plasma levels of TGF-β1 are closely correlated with the severity of renal dysfunction in DN patients [13]. Further studies reveal those patients with DN also develop high urinary levels of TGF-β [14], suggesting the involvement of renal TGF-β in the pathogenesis of DN. This is also found in various animal models of DN [15]. Taken together, these clinical, pathological, and epidemiological findings from both diabetic patients and animal models suggest TGF-β1 may have a pathogenic role in DN, which is to be discussed in this review.

## 2. TGF-β Signaling

TGF-β is a member of the TGF-β superfamily, which also includes bone morphogenic proteins (BMPs), nodals, growth and differentiation factors (GDFs), and activins [16]. TGF-β ligands contain 3 isoforms, TGF-β1, 2, and 3, which are widely expressed in various cell and tissue types with TGF-β1 as the predominant one [17]. TGF-β ligands are synthesized as a larger precursor protein whose N-terminal part is cleaved to release the mature C-terminal ligand in the form of homodimers. The cleaved N-terminal peptide (latency-associated peptide, LAP) physically binds with the C-terminal ligand [16]. The activity of the mature TGF-β homodimers is sequestered by the latent TGF-β-binding proteins (LTBPs), which is termed latent TGF-βs [16]. Active TGF-βs can be released by enzymatic digestion or acid microenvironment [16].

**Figure 1:**
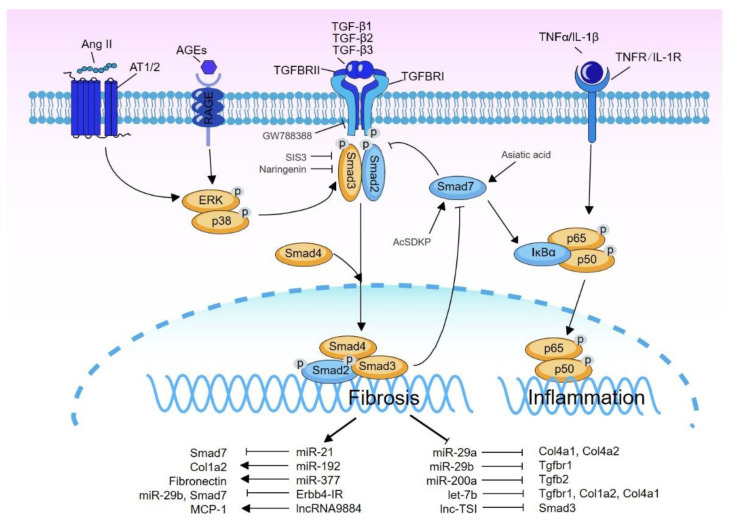
The regulatory role of TGF-β signal in DN. TGF-β ligands (TGF-β1/2/3) transduce the transmembrane signal through binding to TGFBR2 and TGFBR1, resulting in the phosphorylation of downstream Smad2/3. Phosphorylated Smad2/3 form the complexes with Smad4 and translocate into the nucleus to regulate the transcription of target genes. Smad2/3 can also be activated by signal crosstalk with ERK/p38 MAPK pathway. Smad7, an inhibitory Smad, acts to inhibit Smad2/3 phosphorylation by targeting TGFBR1. In addition, Smad7 also induces IκBα, an NK-κB inhibitor, to suppress NF-κB signaling. TGF-β also induces many Smad3-dependent miRNAs/lnRNAs to regulate DN, among which miR-21, miR-192, miR-377, Erbb4-IR, and lncRNA9884 are upregulated and pathogenic. However, miR-29a/b, miR-200a, let-7b, and lnc-TSI are downregulated and renoprotective. The TGF-β signal-targeted inhibitors or agonists, whose efficacy for DN treatment has been validated in animal studies, are also labeled in the illustration. The line with an arrow end means positive regulation, while that with the blunt end means negative regulation or inhibition.

After being released from LAP, active TGF-β binds to the type 2 transmembrane receptor TGFBR2 which is a serine/threonine kinase to recruit and activate TGFBR1 (also called activin receptor-like kinase 5, ALK5) by phosphorylation. Activated TGFBR1 then phosphorylates the receptor-regulated Smads (R-Smad), herein Smad2 and Smad3, at their C-terminal serine residues. Smad2 and Smad3 are the executive transcriptional factors and share high similarities in terms of the amino acid sequence. The activated R-Smads can form complexes with Smad4 (common Smad or Co-Smad) and translocate into the nucleus to regulate the transcription of target genes. There is also an inhibitory Smad7 which is induced by Smad3 and competitively binds to the TGFBR1 to inhibit the phosphorylation of Smad2 and Smad3 [18]. Furthermore, Smad7 can also function to degrade TGFBR1 by recruiting the E3 ubiquitin ligase Smurf2 [19]. Thus, Smad7 negatively modulates TGF-β signaling via its negative feedback mechanism [20] (Figure 1).

The binding of active TGF-β ligand to the receptors is also facilitated by the membrane-bound auxiliary coreceptors betaglycan (TGFBR3) and endoglin [16]. Betaglycan presents TGF-β ligand to TGFBR2 to form a ternary complex, thereby enhancing the responsiveness of TGF-β signaling [21]. Betaglycan can also shed from the membrane to form soluble betaglycan. In contrast to the membrane-bound betaglycan, the soluble betaglycan sequesters the TGF-β signal activity [22]. In addition to the signal transduction from TGF-β ligand to the membrane-bound receptor, Smad2/3 can also be activated/phosphorylated by crosstalk with ERK/p38 MAPK pathway [23].

Furthermore, TGF-β1 can also activate a wide variety of Smad-independent pathways (known as non-Smad signaling) to exhibit its bioactivities. These non-Smad pathways include TGF-β-activated kinase 1 (TAK1), phosphatidylinositol 3-kinase/AKT, and Rho-like GTPase signaling pathways as previously described [24,25]. The activation of these Smad-independent pathways can function alone or synergistically with Smad signaling to regulate the downstream cellular response. One example is that TGF-β1-bound TGFBR1 directly phosphorylates the cell polarity protein Par6, which recruits E3 ubiquitin ligase Smurf1 to degrade the Small GTPase RhoA to initiate epithelial-to-mesenchymal transition (EMT) [26], indicating the complexity of TGF-β signaling.

## 3. Activation of TGF-β Signaling in DN

In both patients and animal models with DN, the TGF-β ligands, TGFBRs, and downstream signaling molecules such as Smad2 and Smad3 are highly upregulated or activated in glomeruli, tubules, and tubulointerstitium [15,23,27,28,29,30,31]. DN is associated with multiple hazardous ambient factors such as high glucose, advanced glycation end products (AGEs), hypertension, and dyslipidemia, which can activate TGF-β signaling through TGF-β-dependent and independent mechanisms.

High glucose potentiates TGF-β signaling and enhances the transcriptional activity of *fibronectin* promoter and *luciferase* construct containing Smad binding elements (SBEs) in the mesangial cell [31]. Indeed, high glucose stimulates the transcription of *Tgfb1* in various kidney cell types including mesangial cell [32,33], fibroblast [34], and proximal tubular cell [35]. The influence of glucose on *Tgfb1* expression may be associated with a putative glucose-responsive element found in the promoter of *Tgfb1* gene [33]. In addition to promoting *Tgfb1* expression, high glucose also enhances the activity of TGF-β1 by up-regulating thrombospondin 1 (TSP1), which can activate the latent TGF-βs [36,37]. Furthermore, high glucose is also found to increase *Tgfbr2* transcription in the murine mesangial cell independent of the induction of TGF-β [29]. Thus, high glucose can activate the TGF-β signaling during the development of DN.

AGEs are produced by the irreversible glycosylation of proteins and ubiquitously distributed in local tissues including kidney in diabetic condition [38,39]. AGEs play a critical role in the pathogenesis of DN, as inhibition of their production significantly attenuates the severity of DN [40]. AGEs can activate multiple intracellular signals including the TGF-β/Smad pathway. In vitro evidence shows that AGEs activate TGF-β/Smad signaling in binary phases in renal tubular cells and vascular cells. In the early phase, AGEs rapidly phosphorylate Smad2/3 within 30 min. This rapid activation of Smad2/3 induced by AGEs is independent of the expression of TGF-β and TGFBRs, but is mediated via the receptor of AGE (RAGE)-ERK/p38 MAKPs-Smad crosstalk mechanism, as the antibody blockade of RAGE or inhibition of ERK/p38 MAPKs abolishes AGEs-induced phosphorylation of Smad2/3. In the later phase, AGEs induce the second peak of Smad2/3 phosphorylation after 24 h which is dependent on the TGF-β and TGFBRs. Importantly, the pharmaceutic inhibition of ERK/p38 MAPKs or over-expression of Smad7 prevents AGEs-induced collagen production, suggesting the critical role of RAGE-ERK/p38 MAKPs-Smad crosstalk pathway in AGEs-induced fibrogenesis in DN [39,41] (Figure 1).

Similar to AGEs, angiotensin II (Ang II) also induces a rapid phosphorylation of Smad2/3 through the Ang II receptor AT1-ERK/p38 MAPKs-Smad crosstalk pathway, which is also independent of TGF-β in vascular smooth muscle cells (VSMCs) and in renal tubular cells [42,43,44]. However, the long-term (24 h) of Smad2/3 activation induced by Ang II requires the *de novo* synthesis of TGF-β [43,44], which is TGF-β-dependent. Furthermore, it is also reported that the activation of protein kinase C is essential for Ang II-stimulated TGF-β1 transcription [45,46].

Hyperlipidemia is an independent risk factor for the development of DN [47]. A recent study indicates that treatment with free saturated fatty acid palmitate in human glomerular mesangial cells causes the activation of Smad2/3 and expression of ECM genes through a CD36-TRPC6-NFAT2 signal axis [48].

In addition to the above mechanisms, reactive oxidative species (ROS) is another factor involving the activation of TGF-β signaling in DN. Indeed, hyperglycemia, Ang II, AGEs, and hyperlipidemia (palmitate) can provoke intracellular ROS overproduction [38,49]. ROS can activate the Activated Protein-1 (AP-1), a transcriptional factor to promote *Tgfb1* expression. Mutation of the AP-1 binding site in *Tgfb1* promoter or pharmaceutically attenuating ROS level reduces the production of TGF-β1 in mesangial cells [50]. Therefore, ROS promotes TGF-β1 expression in an AP-1-dependent mechanism.

## 4. Diverse Role of TGF-β/Smad Signaling in DN

### 4.1. Active Versus Latent TGF-β1 in DN

It is well known that TGF-β is secreted as a latent form but becomes active after it is released from the LAP. The causal relationship between active TGF-β1 and DN is proven in genetically modified mouse models. Transgenic expression of active TGF-β1 specifically in mouse hepatocyte (driven by *Albumin* promoter) causes an elevated level of the circulating active TGF-β1. These mice develop progressive renal failure characterized by mesangial expansion, tubulointerstitial fibrosis, and glomerulosclerosis [51]. Similarly, another study reported that transgenic overproduction of the active TGF-β1 in juxtaglomerular apparatus locally in the kidney also causes renal injury with albuminuria, polyuria, increased ECM deposition in glomeruli, and reduced GFR [52,53]. Thus, these animal studies prove that high levels of active TGF-β1 are pathogenic and can cause renal injury no matter the source if is extrarenal or intrarenal.

By genetically modifying the 3′UTR of *Tgfb1*, the mouse model with transgenic *Tgfb1* expression from 10% (hypomorph) to 300% (hypermorph) over the normal level has been successfully established [54]. After crossing with Akita diabetic mice, mice with *Tgfb1* hypomorph are prevented from the development of DN while mice with *Tgfb1* hypermorph develop severe DN. Furthermore, *Tgfb1* expression was also conditionally modified in podocyte and proximal tubule in Akita mouse. Interestingly, the switching of *Tgfb1* expression from hypomorph to hypermorph in podocyte causes glomerular injury by a 4-fold increase in albuminuria. However, the switching of *Tgfb1* from hypomorph to hypermorph in proximal tubule results in tubulointerstitial fibrosis, polyuria, glucosuria, and macroalbuminuria (increase by 20 folds) [15]. These findings suggest that TGF-β1 may play a distinct role in different segments of the nephron in DN. The association of overexpressing tubular TGF-β1 with the development of albuminuria, polyuria, and glucosuria in DN may be due to the impairment of renal reabsorption as *Tgfb1*-hypermorphic Akita mice show decreased expression of megalin [15]. This is also supported by the in vitro findings that TGF-β1 is able to suppress the glucose uptake by decreasing the expression of glucose transporters sodium-glucose co-transporter 1/2 (SGLT1/2) and to inhibit albumin reabsorption by reducing megalin expression in cultured proximal tubular cells [55,56].

In contrast to active TGF-β, latent TGF-β1 is protective in DN. Mice with transgenic overexpression of latent TGF-β1 specifically in skin epidermis show normal renal phenotype despite a 10-fold increase in circulating latent TGF-β1. However, these transgenic mice are protected from the development of renal inflammation and renal fibrosis in models of obstructive nephropathy and crescentic glomerulonephritis [57]. Our recent observation also found that overexpression of latent TGF-β1 in the skin is capable of inhibiting STZ-induced DN (unpublished data). The distinct roles of latent versus active TGF-β1 are also reflected in the regulation of immune and inflammatory responses. TGF-β1 is an anti-inflammatory cytokine as *Tgfb1*-null mice develop multiple organ inflammation and die prematurely [58]. By contrast, overexpression of latent TGF-β1 in the epidermis results in a marked activation of TGF-β/Smad signaling and the development of inflammatory skin lesions locally with massive infiltration of immune cells [59]. These observations suggest that the function of latent TGF-β1 is disease and environment-dependent although it is generally protective in kidney disease. Thus, clinical treatment using the antibody-based strategies should be cautious since most circulating TGF-β1 is in latent form, and administration of anti-TGF-β antibody may block the beneficial effect of latent TGF-β1 and result in an adverse effect on patients with DN.

TGF-β signaling also plays a regulatory role in renal inflammation. TGF-β can activate NLRP3 inflammasomes, which is important for the maturation and release of inflammatory cytokines such as IL-1β and IL-18, in a Smad3-dependent manner [60,61]. In turn, NLRP3 also enhances TGF-β signaling by promoting TGF-β1-induced Smad3 phosphorylation during the process of EMT [61]. Thus, NLRP3 deficiency attenuates TGF-β1-triggered EMT and renal fibrosis in vitro and in diabetic mouse model [60,61,62,63].

### 4.2. TGF-β Receptors

As the binding receptor of TGF-β ligands, TGFBR2 is important for the transmission of downstream Smad signaling. Dominant-negative TGFBR2 abolishes the activation of canonical TGF-β/Smad signal and fibrosis in VSMCs in response to Ang II [43]. In CKD, conditional deletion of *Tgfbr2* in tubules attenuates renal fibrosis in UUO mice [64]. Similarly, heterozygous deletion of *Tgfbr2* (*Tgfbr2*+/−) reduces the severity of glomerular hypertrophy and mesangial expansion in STZ-induced DN [65]. However, TGFBR2 plays complex roles in the regulation of inflammation. Deletion of *Tgfbr2* in the tubule aggravates renal inflammation in UUO mice while alleviating inflammation in cisplatin-induced AKI [64,66]. Therefore, the regulatory role of TGFBR2 in inflammation may be disease type-dependent. Although there is no available genetic data for the role of TGFBR1 in kidney disease including DN, inhibition of TGFBR1 by the specific pharmaceutical inhibitors IN-1130 and SB-525334 can inhibit fibrosis in the rat models of UUO and puromycin aminonucleoside (PAN)-induced nephritis, respectively [67,68].

In addition to TGFBRI/II, the TGF-β co-receptors betaglycan and endoglin also play critical roles in DN. The soluble betaglycan can sequester TGF-β from its receptor and negatively modulate the signal activity [21]. It is reported that treatment with recombinant soluble betaglycan improves renal injury in db/db mice [69]. Endoglin is highly expressed in the kidney biopsy of patients with DN and positively correlates with renal dysfunction [69]. Knockdown of endoglin mitigates TGF-β1-stimulated fibrosis in human fibroblast cells [70], suggesting a deleterious role for endoglin in DN.

### 4.3. Smad3 vs. Smad2

Smad2 and 3 share the same DNA binding motif or Smad-binding elements (SBEs) with a sequence of “AGAC” [27]. However, Smad2 and 3 usually target different subsets of genes [27]. Furthermore, because of the short DNA recognition motif, Smad2 and Smad3 have low DNA binding affinity and specificity. Therefore, they should interact with other partner proteins or transcriptional factors to establish efficient recognition (or binding) to the cis-regulatory element of their target genes [28]. These binding partners largely determine the targeting specificity and transcriptional regulation fashion (promotion or repression) in a context- and cell type-dependent manner [28].

Increasing evidence shows that Smad3, but not Smad2, is the primary profibrotic transcriptional factor in response to the various fibrogenic mediators including TGF-β [31], Ang II [43,44], and AGEs [41]. In the mouse model of UUO, deletion of Smad3 significantly reduces renal fibrosis [71]. By contrast, Smad2 is protective as knockout of Smad2 in tubular cells exacerbates fibrosis in UUO mice, which is associated with increased Smad3 activity [72]. Unlike fibrosis, both Smad2 and Smad3 show similar activity on inflammation, as knockout of Smad2 or Smad3 attenuates inflammatory injury in acute kidney disease (AKI) [66,73]. In view of DN, mice null for Smad3 are protected from renal fibrosis including GBM thickening and ECM overproduction in STZ-induced DN [74], although inhibition of albuminuria is always observed [75]. In type 2 diabetes-associated DN, our study reveals that deletion of Smad3 from db/db mice prevents the development of DN as Smad3KO-db/db mice are free from diabetes and DN with normal levels of blood glucose and serum creatinine without insulin resistance, glucose intolerance, obesity, albuminuria, and renal pathology [76,77]. All these findings demonstrate an essential role for Smad3 in the pathogenesis of DN in both type 1 and type 2 diabetes. Interestingly, in contrast to the anti-fibrogenic role of Smad2 in UUO mouse kidney [72], conditional deletion of Smad2 from fibroblasts driven by the Fibroblast-specific Protein 1 (*Fsp1*) promoter reduces renal fibrosis in STZ-induced DN [78]. As *Fsp1* promoter is also active in hematopoietic cells [79], more studies are needed for a better understanding of the role of Smad2 in DN.

### 4.4. Smad4

Although Smad4 is a common Smad to facilitate the nuclear translocation of Smad2/3, it seems to play diverse roles in CKD including DN. It has been shown that knockout of Smad4 promotes inflammation but inhibits fibrosis in the kidney of UUO mice, which is associated with reduced Smad7 expression and decreased Smad3 activity, thereby enhancing the inflammatory NF-κB signaling but inactivating the fibrotic Smad3 signaling [80].

Smad4 also plays a profibrotic role in DN. It is reported that knockdown of Smad4 by injecting the locked nucleic acid (LNA) can ameliorate glomerulosclerosis and albuminuria in *eNOS*−/− mice fed with high-fat diet (HFD) without influencing the diabetic phenotype [81]. This renoprotective effect is further recapitulated in HFD-fed *eNOS*−/− mice with podocyte-specific deletion of Smad4. Mechanistically, deletion of Smad4 from podocytes is able to enhance glycolysis and oxidative phosphorylation as Smad4 can bind to PKM2 and ATPase inhibitory factor 1 (ATPIF1) to inhibit glycolysis and oxidative phosphorylation [80].

### 4.5. Smad7

Smad7 plays a pivotal role in anti-fibrotic response to TGF-β by inhibiting Smad2/3 phosphorylation through competing for TGFBR1 binding and inducing its degradation [18,19]. In vitro, overexpression of Smad7 inhibits CTGF expression and ECM synthesis induced by AGEs and Ang II in renal tubular cells and VSMCs [39,41,42,44]. *In vivo*, overexpression of Smad7 attenuates fibrosis in different renal disease models including 5/6 nephrectomy [82], crescentic glomerulonephritis [83], UUO [84], chronic aristolochic acid nephropathy [85], and hypertensive nephropathy [86]. By contrast, disruption of Smad7 aggravates renal fibrosis in the UUO kidney [87], aristolochic acid nephropathy [85], and hypertensive nephropathy [88]. In addition to inhibiting fibrosis, Smad7 also suppresses inflammatory NF-κB signaling by inducing the expression of IκBα [89]. To support this notion, overexpression of Smad7 can inhibit renal inflammation in animal models of crescentic glomerulonephritis [83], 5/6 nephrectomy [90], chronic aristolochic acid nephropathy [85], and hypertensive nephropathy [86]. Inversely, knockout of Smad7 promotes inflammation with more severe impairment of renal function in chronic aristolochic acid nephropathy [85], hypertensive nephropathy [88], and UUO kidney [87].

Smad7 also plays a protective role in DN. In STZ-induced DN and db/db mice-associated DN, Smad7 knockout mice develop more severe albuminuria, renal fibrosis (glomerulosclerosis, tubulointerstitial ECM production), and inflammation (macrophage infiltration, expression of inflammatory cytokines such as TNF-α, IL-1β, and MCP-1) [91,92]. However, ultrasound microbubble-mediated delivery of Smad7 into the kidney of db/db mice ameliorates the renal fibrosis and inflammation accompanied by improved renal function [92].

## 5. Role of TGF-β/Smad3-Dependent miRNAs and Long Non-Coding RNAs in DN

miRNA and long non-coding RNA (lncRNA) are two important subclasses of the non-coding RNA family and have been shown to play critical roles in various physiological and pathological processes through distinct mechanisms. miRNAs are short single-stranded RNAs with a length of 20–22 nucleotides, and they function by binding to the 3′ untranslated region (UTR) of target mRNA to induce translational repression or degradation [93]. In contrast to miRNAs, lncRNAs have a size of more than 200 nucleotides and are engaged in the transcriptional regulation of neighboring or non-neighboring genes with distinct mechanisms. lncRNAs may also function by directly interacting with particular proteins to regulate protein-specific roles (for a detailed review, see reference [94]). Accumulating evidence suggests that miRNAs and lncRNAs are functionally engaged in TGF-β/Smad signal-driven kidney disease [89,95]. High-throughput microarray and transcriptome analysis revealed that a number of miRNAs and lncRNAs are differentially expressed in the diseased kidney under the regulation of TGF-β signaling [96,97,98,99,100]. Among them, Smad3-dependent miR-21, miR-192, miR-377, Erbb4-IR, and lncRNA9884 are upregulated and proven to be pathogenic, while miR-29a/b and miR-200a are downregulated and renoprotective in DN (Figure 1 and Table 1).

### 5.1. miRNAs

miR-21 is a Smad3-dependent miRNA and is upregulated in various kidney diseases including DN [98,116]. Knockdown of miR-21 alleviates albuminuria and reduces renal fibrosis and inflammation in db/db mice [101]. Mechanistically, miR-21 directly targets the 3′UTR of Smad7 mRNA, therefore suppressing the anti-fibrotic and anti-inflammatory functions of Smad7 [101].

miR-192 is also a Smad3-dependent miRNA and is upregulated in STZ-induced DN and db/db mice-associated DN [103]. The functional study demonstrates that miR-192 mediates renal fibrosis by targeting the Smad-interacting protein 1 (*SIP1*), which is an E-box repressor to transcriptionally suppress *Col1a2* [102]. Therefore, miR-192 indirectly upregulates *Col1a2* via attenuating *SIP1* expression. Knockdown of miR-192 ameliorates renal fibrosis in STZ-induced DN in mice [117]. In patients with type 2 diabetic nephropathy (T2DN), serum levels of miR-192 are elevated [118]. However, other studies also reported decreased miR-192 in renal biopsies and serum in patients with T2DN [105,119]. In human proximal tubular HK2 cells, TGF-β1 downregulates miR-192, and overexpression of miR-192 suppresses TGF-β1-induced E-cadherin loss, suggesting a protective role against epithelial to mesenchymal transition (EMT) [119]. It is not clear whether this discrepancy is caused by species variance in these studies.

miR-377 is upregulated in mesangial cells stimulated with high glucose or TGF-β1, and in kidneys of STZ-induced diabetic mice and diabetic NOD mice. Furthermore, increased serum miR-377 is detected in patients with T2DN [105]. Importantly, miR-377 promotes fibronectin expression in mesangial cells, although the direct target gene remains to be elusive [104].

The miR-29 family includes three family members (miR-29a,b,c) and is made up of Smad3-dependent miRNAs [99]. Among them, miR-29b is well characterized by its anti-fibrotic property in multiple organs [99,109,120,121]. miR-29b is downregulated in the kidney of db/db mice and in AGE-treated mesangial cells. Overexpression of miR-29b relieves albuminuria accompanied by attenuated renal fibrosis and inflammation in db/db mice [108]. Mechanistically, miR-29b inhibits *Tgfbr1* expression by targeting its coding sequence (3rd exon) [109]. Another member of the miR-29 family, miR-29a, is also downregulated by high glucose in the proximal tubular cells (HK2) [106]. Transgenic overexpression of miR-29a ameliorates renal hypertrophy, fibrosis, and inflammation in STZ-induced DN [107]. Further analysis demonstrates that miR-29a directly binds to the 3′UTR of both *Col4a1* and *Col4a2* to downregulate their expression [106].

miR-200 family is downregulated by TGF-β1/2. Among them, miR-200a is downregulated in STZ-induced DN in ApoE-knockout mice. Overexpression of miR-200a suppresses fibrosis and EMT phenotype in the tubular cell line NRK53E. It has been shown that miR-200a directly binds to the 3′UTR of *Tgfb2* to decrease its expression [113]. Thus, the miR-200 family may be protective in DN.

miRNA let-7b is also downregulated in STZ-induced DN and in tubular and mesangial cells in response to TGF-β1. Overexpression of let-7b attenuates fibrosis by negatively regulating *Tgfbr1*, *Col1a2,* and *Col4a1* through direct binding to their 3′UTR [114,115]. miRNAs from the let-7 family are also upregulated by fibroblast growth factor receptor (FGFR) signaling and are important to maintain the quiescence of endothelia. Activation of TGF-β signaling causes endothelial-to-mesenchymal transition (EndoMT) with decreased anti-fibrotic miR-29s and let-7s [122]. It is reported that miR-29s can upregulate let-7s by directly targeting INF-γ to enhance FGFR signaling, therefore creating a crosstalk between miR-29s and let-7s [123]. However, the in vivo role of let-7b in DN remains unexplored.

### 5.2. lncRNAs

By using RNA sequencing, many Smad3-dependent lncRNAs have been identified and found to play critical roles in renal fibrosis and inflammation [100]. Among them, lncRNA Erbb4-IR (np_5318) is upregulated in DN of db/db mice and in AGEs-stimulated mesangial cells in a Smad3-dependent manner. Kidney-targeted silencing of Erbb4-IR reduces albuminuria, serum creatinine, and renal fibrosis in db/db mice. The mechanistic study reveals that Erbb4-IR binds to the 3′ region of miR-29b genomic locus to suppress its transcription [111]. Furthermore, in the UUO kidney Erbb4-IR is found to promote fibrosis through inhibiting Smad7 transcription by binding to its corresponding genomic sequence of 3′UTR [110]. Therefore, Erbb4-IR functions as a trans-regulator to negatively regulate miR-29b and Smad7 in renal fibrosis.

lncRNA9884 is another Smad3-dependent lncRNA. lncRNA9884 is upregulated in db/db mice and AGE-treated renal tubular cells. lncRNA9884 promotes inflammation by binding to the promoter of *MCP-1* gene to enhance its transcription [112]. Knockdown of lncRNA9884 attenuates albuminuria and serum creatinine by inhibiting renal inflammation.

A recent study found a novel lncRNA lnc-TSI (TGF-β/Smad3-interacting long non-coding RNA) whose expression is Smad3-dependent. lnc-TSI directly binds to the MH2 domain of Smad3 protein to block its interaction with TGFBR1 and the resulted phosphorylation. Delivery of lnc-TSI into the kidney attenuates renal fibrosis in UUO mice. Furthermore, the expression level of lnc-TSI is negatively correlated with renal fibrosis in patients with IgA nephropathy (IgAN). Thus, lnc-TSI functions to repress TGF-β/Smad signaling via a negative feedback mechanism similar to, but independent of, Smad7 [124]. However, the role of lnc-TSI in DN remains unclear.

## 6. Treatment of DN by Targeting TGF-β Signaling

During the last decades, many treatment strategies have been developed and proven to effectively ameliorate DN clinically. Many drugs targeting diabetes by lowering blood glucose reasonably benefit renal function in DN, e.g., SGLT2 inhibitors empagliflozin [125] and canagliflozin [126], and DPP-4 inhibitor linagliptin [127]. Furthermore, targeting DN-associated risk factors such as hypertension with angiotensin-converting enzyme inhibitors (ACEis) [128] and angiotensin II receptor blockers (ARBs) [129], and hyperlipidemia with statins [130] also significantly delays the progression of DN. In addition, the pharmaceutical intervention of particular pathways such as mineralocorticoid receptor signaling [131], endothelin A receptor signaling [132], JAK-STAT signaling [133], and glycolysis [134] is also proven to effectively improve renal function in DN. However, all these treatments have been shown to delay, but cannot prevent, the progression of DN. Therefore, more effective therapeutic drugs for diabetes are urgently needed.

Given its critical roles of TGF-β/Smad signaling in the development of DN, TGF-β signal-targeted therapy seems to be promising for the treatment of DN. It is reported that blockade of TGF-β signaling at levels of ligands and receptors by neutralizing antibody or soluble TGF-β receptor (TGFBR2) is effective to relieve DN [56,135,136,137]. However, a phase 2 clinical trial shows that treatment with a TGF-β1-neutralizing antibody has no benefit to patients with DN [138], suggesting that the antibody-based therapy may not be a good strategy for the treatment of DN. Nevertheless, the failure of TGF-β antibody treatment remains largely unexplained. It is possible that blockade of TGF-β1 by treatment with the anti-TGF-β antibody may promote inflammation as TGF-β1 is a potent anti-inflammatory cytokine [139]. It is also possible that the antibody-based treatment may also block the latent TGF-β1 and thus blunt its protective effect on DN. In addition, the prolonged treatment with the anti-TGF-β1 immunoglobin for 8 months may also cause autoantibody production [140], which in turn may reduce the therapeutic effect of anti-TGF-β1 antibody treatment on DN. Therefore, more well-designed clinical studies are needed to assess the efficacy of neutralizing antibodies against TGF-β ligand in the treatment of DN.

Targeting TGF-β receptors may be an alternative strategy for the treatment of DN. GW788388, an inhibitor of both TGFBR1 and TGFBR2, has been proven curative to attenuate renal fibrosis in db/db mice [141]. It is possible that several other chemical inhibitors to TGFBR1 (ALK5) may also have therapeutic effects on DN. However, no animal experimental data so far are available. Another potential therapeutic agent functioning at the receptor level is the soluble coreceptor betaglycan. The animal study demonstrated that administration of soluble betaglycan suppresses renal fibrosis in db/db mice [69]. However, the clinical significance of soluble betaglycan remains to be determined.

It is reported that the DPP-4 inhibitor linagliptin can suppress the conversion of latent TGF-β into its active form and shows anti-fibrotic ability in the STZ-induced DN, indicating a renoprotective role independent of its glucose-lowering effect [127,142]. Furthermore, DPP-4 also interacts with integrin β1 to enhance the heterodimerization of TGF-β receptors and the responsiveness of TGF-β signaling [122]. Therefore, DPP-4 inhibitors are renal protective in DN by suppressing the TGF-β signaling at both the ligand and receptor levels. SIRT3 is also renal protective in DN as SIRT3 deficiency promotes abnormal glycolysis and renal fibrosis by activating TGF-β/Smad signaling, which is reversed by restoring SIRT3 [134], revealing SIRT3 as a potential therapeutic agent for DN.

As Smad3, but not Smad2, plays a critical role in the pathogenesis of diabetes and DN [76,77], targeting Smad3 represents a novel and effective strategy for the treatment of DN. In the UUO mouse model, inhibition of Smad3 with specific inhibitor of Smad3 (SIS3) attenuates renal fibrosis and inflammation [143,144]. It is also reported that treatment with SIS3 can block EndoMT and improve renal dysfunction and fibrosis in STZ-induced DN [145]. We also show that treatment with SIS3 protects against DN in db/db mice by preventing lysosome from depletion and thus improves the autophagic flux in the renal tubular epithelial cells [146]. However, more data are needed to evaluate the efficacy and safety of SIS3 in other animal models of DN. Interestingly, the glucocorticoid receptor (GR) signaling also plays a protective role in DN. A recent study showed that endothelial deletion of GR accelerates fibrosis in DN by enhancing EndoMT [147]. Mechanistically, GR suppresses TGF-β/Smad3 signaling by physically interacting with Smad3 protein and inhibiting its activity [148]. Therefore, GR may serve as an inhibitor of Smad3. However, the GR agonist dexamethasone shows unfavorable side effects to exacerbate hyperglycemia [147].

Smad7 is another ideal therapeutic agent for the treatment of DN as it can suppress both TGF-β/Smad3-mediated fibrosis and NF-κB-mediated inflammation simultaneously (Figure 1) [92]. Overexpression of Smad7 through the kidney-targeted gene delivery by ultrasound microbubble technique improves renal function in different animal models of DN [91,92]. However, exogenous DNA delivery-based therapy may cause a safety concern for clinical application. Thus, developing Smad7 agonists may be an alternative for treatment of DN. One promising option is N-acetyl-seryl-aspartyl-lysyl-proline (AcSDKP). AcSDKP is an endogenous anti-fibrotic peptide and orally appliable. Treatment with AcSDKP has been shown to have a significant renoprotective effect on DN in both type 1 and 2 diabetic mouse models [149,150]. The mechanistical study reveals that AcSDKP may serve as a Smad7 agonist by promoting its cytoplasmic translocation, which happens without TGF-β stimulation [151] (Figure 1). However, clinical trials to assess the efficacy of AcSDKP in patients with DN remain to be investigated.

Furthermore, targeting Smad3-dependent miRNAs and lncRNAs by kidney-specifically overexpressing protective or silencing pathogenic miRNAs/lncRNAs may be a specific strategy for the treatment of DN. However, Smad3-dependent miRNAs/lncRNAs gene therapy also faces the same situation as Smad7 gene therapy does. Thus, the development of Smad3-miRNAs/lncRNAs specific antagonists or agonists should have therapeutic potential clinically.

Traditional Chinese medicine (TCM) or complementary and alternative medicine could be an alternative approach for the treatment of DN. TCM has long been used for the treatment of many diseases including diabetes in Asian countries. TCM is usually sourced from herbs and contains multiple pharmaceutically active ingredients which can target different disease-related signaling pathways simultaneously [152]. The long-term benefits of TCM for patients with CKD including DN have been validated in a large cohort retrospective study [153]. For example, the TCM prescriptions Tangshen formula [154,155] and Chaihuang-Yishen granule [156] have been shown to effectively inhibit TGF-β/Smad3 signaling and reduce renal fibrosis in several experimental models of DN. Berberine, an herbal extract derived from *Cortex Phellodendri Chinensis*, shows inhibitory activity on TGF-β/Smad3 signaling and attenuates renal fibrosis and inflammation in db/db mice [157]. We also found that unbalanced TGF-β/Smad3/Smad7 signaling is a key mechanism of DN and rebalancing this pathway by treatment with purified products from TCM can effectively inhibit DN. For example, we showed that asiatic acid (from *Centella asiatica*) is a Smad7 agonist, and naringenin (a flavonoid rich in fruits of citrus family) functions as a Smad3 inhibitor. The combination of asiatic acid and naringenin can synergistically inhibit Smad3 while increasing Smad7 to rebalance the TGF-β/Smad signaling and alleviate renal fibrosis in UUO mice [158]. Furthermore, our unpublished data also demonstrate a renoprotective role of the compound of asiatic acid and naringenin against DN in db/db mice, suggesting rebalancing TGF-β/Smad signaling by TCM may be an innovative approach for better treatment of DN.

## 7. Conclusions and Perspectives

TGF-β/Smad signaling is a key pathway in the pathogenesis of DN. However, the roles of TGF-β1 in DN are complicated and distinct. TGF-β diversely regulate DN via its ligands, receptors, and downstream Smad molecules. In diabetes-induced renal fibrosis and inflammation, latent TGF-β1 and Smad7 are protective but active TGF-β1 and Smad3 are pathogenic. TGF-β/Smad signaling also diversely regulates DN by upregulating or downregulating a number of Smad3-dependent miRNAs/lncRNAs to either positively or negatively regulate the development of DN. Among these non-coding RNAs, miR-29a/b, miR-200a, and let-7b families are protective while miR-21, miR-192, miR-377, Erbb4-IR, and lncRNA9884 are pathogenic. Thus, targeting TGF-β/Smad signaling by specifically rebalancing Smad3/Smad7 signaling with either Smad3 inhibitors and/or Smad7 agonists and Smad3-dependent miRNAs/lncRNAs related to renal fibrosis and inflammation could be a better therapeutic strategy for combating DN.

Although the in vivo and in vitro studies have provided profound evidence to prove the roles of TGF-β signaling in the pathogenesis of DN, there are still many concerns with respect to the specificity, complexity, and diversity of TGF-β signaling in DN. Firstly, the cell type-specific roles of TGF-β signaling have been perceived in the study of TGF-β1. Many functional studies have been conducted in animals with conventional gene knockout or conditional gene knockout in limited renal cell types. Therefore, more intensive investigations with different cell type-specific genetical modifications are needed for the full understanding of TGF-β signaling in DN. Secondly, the transcriptional regulation of Smad-dependent genes that are specifically related to fibrosis or inflammation needs to be further investigated. This is important because the identification of precise mechanisms related to renal fibrosis and/or inflammation is the first step towards the development of more specific therapy for DN. Finally, although genetical and pharmaceutical studies demonstrate that intervention of TGF-β signaling is promising for the treatment of DN, currently no TGF-β signal-targeted drugs are available for clinical application. Therefore, more studies are needed for the development of innovative drugs to treat DN by rebalancing TGF-β signaling. Considering its complexity and diversity, TCM could be a promising resource for the screen and development of innovative drugs for the better treatment of DN.

## Figures and Tables

**Table 1:** TGF-β/Smad3-dependent miRNAs and lncRNAs with validated roles in DN.

miRNA/lncRNA	ND Model	Target Gene and Reference
Pathogenic and upregulated		
miR-21	db/db mice	Smad7 [101]
miR-192	STZ-induced DN db/db mice	Col1a2 via directly targeting SIP1 [102,103]
miR-377	STZ-induced DN diabetic NOD mice	unclear [104,105]
miR-29a	STZ-induced DN	Col4a1 and Col4a2 [106,107]
miR-29b	db/db mice	Tgfbr1 [108,109]
Erbb4-IR	db/db mice	miR-9b, Smad7 [110,111]
lncRNA9884	db/db mice	MCP-1 [112]
Renoprotective and downregulated		
miR-200a	STZ-induced DN	Tgfb2 [113]
let-7b	STZ-induced DN	Tgfbr1, Col1a2, Col4a1 [114,115]

## Data Availability

Not applicable.
